# Eczema Capitis

**Published:** 1884-04

**Authors:** 


### ECZEMA CAPITIS.

Eczema capitis frequently confines itself to the space behind the auricle; in young babies it is often seen in this location. Sometimes it appears in each child of a family as they reach a certain age. Eczema is most frequently a result of some form of mal-assimilation, hence *local* treatment is *pathologically* wrong and clinically useless.

As to the homœopathic remedies which seem to act more especially on this portion of the skin—behind the auricle—Jahr gives us:

BLOTCHES behind the ears: Bry., *Calc.*, Carbo an., Caust., Staph.

DAMPNESS behind the ears: Amm. carb., *Calc.*, Carbo veg., Caust., *Graph.*, Kali carb., *Lyc.*, Nitr. acid, *Oleand.*, *Petr.*, Phosp., Silicea.

ERUPTIONS behind the ears: Ant. crud., *Baryta*, *Calc.*, Canth., Chin., *Cicuta*, Graph., Hepar, Mezer., Oleand., *Puls.*, Sabad., Selen., Sil., *Staph.*

HERPES behind the ears: Amm. m., Graph., *Oleand.*, *Sepia.*

ITCHING behind the ears: Agar., Alum., Carbo veg., *Graph.*, Mosch., Natr. m., Nitr. acid, Therid.

Under *itching*, Allen adds: Aur., Calc., Fago, Hura, Rhus v., Sulph. Tilia, Verat.

Itching, evening in bed: Sulph.; night in bed: Merc. iod. fl.; worse at night: Aur. mur.; worse by scratching: Mag. carb. (r.), Mag. mur. (l.), Ruta (l.); acute: Mezer.; extending to nape of neck: Rhod. (l.); persistent: Lyc.

— as from tetter: Hura.

SCURFS behind the ears: *Graph.*, Hepar, Lyc., Puls., *Staph.*

SORENESS behind the ears: Anac., Cicuta, *Graph.*, Kali carb., *Lach.*, Lyc., Merc., Mur. acid, Nitr. acid, Petr., Psorin.

SWELLING behind the ears: Bry., Calc., Caps., Carbo an., Tabac.

Under head of eruption behind ear, Allen's *Index* gives:

ERUPTION: Guare, Jug. (r.); burning itching, agg. at night, Viola tr.; itching, Mag. sul. (r.); itching after scratching, Mag. m.; resembling itch in children, Arund.; moist, *Calc.;* itching, scurfy, Staph.; sore, Psor. (r.); red, irregular spots, Cocc.

The above gives a *resume* of the local therapeutics; the wise prescriber will by no means confine his attention to the *local symptoms.* Attention to dietetics is a necessary part of the treatment of these cases, especially so with infants.

In cases where the eruption heals readily but breaks open, or is scratched open frequently, the remedy will probably be found in either Graphites or Mezereum. Of Graphites, Guernsey says: "The eruption exudes a transparent, glutinous fluid, *which* causes the crust to fall off, when more form to fall off again in turn; meanwhile the eruption extends over a still larger surface." Of Mezereum, we read: Child continually *scratches* the face, which is covered with blood; itching worse at night; it *tears* off the scabs, leaving spots on which fat pustules form.

